# Allergic rhinitis, asthma and laryngopharyngeal reflux disease: a cross-sectional study on their reciprocal relations

**DOI:** 10.1038/s41598-020-80793-1

**Published:** 2021-02-03

**Authors:** Ameer Kakaje, Mohammad Marwan Alhalabi, Ayham Alyousbashi, Ayham Ghareeb

**Affiliations:** Faculty of Medicine, Damascus University, Damascus, Syria

**Keywords:** Environmental sciences, Medical research, Risk factors, Signs and symptoms

## Abstract

Allergic rhinitis (AR) is a common medical condition worldwide. It is an inflammation in the nasal mucosa due to allergen exposure throughout the year. Laryngopharyngeal reflux (LPR) is another medical condition that can overlap with AR. LPR can be considered an extra oesophageal manifestation of gastro-oesophageal reflux disease (GORD) or a different entity. Its diagnosis imposes a real challenge as it has a wide range of unspecific symptoms. Although AR and LPR are not life-threatening, they can severely affect the quality of life for years and cause substantial distress. Moreover, having AR is associated with having asthma which is also in turn associated with GORD. This is a cross-sectional study which used surveys distributed online on Social Media and targeted people across Syria. All participants who responded to the key questions were included. Reflux symptom index (RSI) was used for LPR, and score for allergic rhinitis (SFAR) was used for AR. Demographic questions and whether the participant had asthma were also included in the survey. We found that there was an association between the symptoms of LPR and AR p < 0.0001 (OR, 2.592; 95% CI 1.846–3.639), and their scores were significantly correlated (r = 0.334). Having asthma was associated with LPR symptoms p = 0.0002 (OR 3.096; 95% CI 1.665–5.759) and AR p < 0.0001 (OR 6.772; 95% CI 2.823–16.248). We concluded that there was a significant association between having LPR, AR, and asthma. We need more studies to distinguish between their common symptoms and aetiologies.

## Introduction

Laryngopharyngeal reflux (LPR) occurs when the reflux of gastric contents reaches the upper aerodigestive tract without having heartburn or regurgitation^1^. LPR can be considered an atypical presentation of gastro-oesophageal reflux disease (GORD) or a different entity^2,3^. In Syria, 31.9% suffered of LPR symptoms^4^. Asthma association with GORD can be explained by the coughing and increased intra-abdominal pressure in asthma which may induce GORD symptoms. On the other hand, gastric reflux can directly damage pulmonary tree, causing bronchoconstriction^5–9^.

Allergic rhinitis (AR) is one of the most common diseases worldwide; it is an inflammatory medical condition that occurs in the nasal mucosa due to allergens exposure^10^. AR prevalence ranges from 5 to 22% worldwide^11^. Moreover, a survey in the Middle East found that 10% of responders had AR^12^. However, AR symptoms were found in around half of the population in one study in Syria^13^. Although AR is not life-threatening, it affects the quality of life and predisposes to multiple airway conditions^14–17^. AR and asthma can be viewed as two corresponding airway diseases as they have common characteristics^18^. AR has a wide variety of symptoms including sneezing, nasal itching, rhinorrhoea, and nasal congestion/obstruction^19,20^. A causal link between GORD and AR was not established, and only a few studies indicated an association^21^. However, as AR and LPR have many symptoms in common from the irritation of the aerodigestive tract, they may be associated with one another which was suggested by many studies regardless of having asthma^22,23^.

In this study, we used reflux symptom index (RSI)^24–28^ to assess LPR symptoms, and score for allergic rhinitis (SFAR)^29,30^ to assess AR symptoms. We aim to determine the association between LPR, AR, and asthma.

## Methods and materials

This is a cross-sectional study that was conducted in Syria. Online surveys were used to cover the largest population possible. Surveys were distributed to different Social Media groups that covered different topics. Demographic questions were asked such as gender, and age. People from across all Syria could participate. We included any person who accepted to participate, lived Syria, and answered key questions. The surveys were posted multiple times during the day in March in 2019. No medical diagnosis or follow-ups were conducted.

### Ethical approval and consent of participants

This study protocol was approved by faculty of medicine Damascus University deanship ethical committee. All methods were performed in accordance with the relevant guidelines and regulation and in accordance with the Declaration of Helsinki. STROBE guidelines were used in this study.

Informed consent was taken for participating in the research, and for using and publishing of the data.

### Measurements

We used a form of RSI which was validated in Arabic^28^. RSI is a self-administered questionnaire which relies on a scoring system for symptoms that evaluates the possibility of LPR^24,25^. RSI is a nine-item scale questionnaire about symptoms suggestive of LPR as shown in the tables. The scale ranges from 0 when answering “no problem” to 5 when answering “severe problem” to each item. The total score ranges from 0 to 45 and the cut-off point was set to 13 or more to suggest the possibility of having LPR-suggestive symptoms.

We also used the Arabic version of SFAR, a simple self-reported tool^29,30^. SFAR is a structured scoring system that has eight questions about symptoms of AR, the personal and familial history of allergy, and allergy tests. These questions are shown in the tables. SFAR score ranges from 0 to 16 and positive answers earn points which can be added, and the cut-off point was set to 7.

We directly asked about having asthma as we could not perform medical diagnose to the participants. The survey included basic demographics, a question whether or not the participant had asthma, and RSI and SFAR questions. We could not follow-up with medical examination or investigation. We could not determine when the symptoms overlap, and therefore bias could not be reduced.

### Data analysis

Data were processed using IBM SPSS software version 26 for Windows (SPSS Inc, IL, USA). Chi-square, independent t-test, and odds ratios (ORs) were used for categorical variables while Pearson correlation coefficient was used for continuous numeral variables. Values of less than 0.05 for the two-tailed p values were considered statistically significant. Any participant with missing data in key questions was eliminated.

### Preprint

A previous version of this manuscript was published as a preprint^31^.

## Results

This study included 673 subjects, of which 170 were males and 503 were females with a mean age of 23.9 ± 6.6 years. It was found that 341 (50.7%) had AR, 212 (31.5%) had LPR symptoms, and 44 (6.5%) had asthma. In subjects with AR, 38 out of 341 (11.1%) had asthma. In subjects with LPR symptoms, 25 out of 212 (11.8%) had asthma. This was demonstrated in (Table 1) along with characteristics of subjects, their demographic data, and RSI and SFAR results and scores. We used chi-square and odds-ratio to compare subjects with negative and positive final results (Table 2) and found that having symptoms suggestive of LPR was associated with having AR p < 0.0001 (OR, 2.592; 95% CI 1.846–3.639).

**Table 1:** Characteristics of subjects, their demographic data, and RSI and SFAR results and scores.

Characteristic	Frequency (n = 673)	Percentage%
**Gender**		
Male	170	25.3
Female	503	74.7
**RSI results**		
Positive AR	341	50.7
Negative AR	332	49.3
**SFAR results**		
Positive LPR symptoms	212	31.5
Negative LPR symptoms	461	68.5
**Having asthma**		
Across all the sample	44	6.5
Only across subjects with AR	38	11.1
Only across subjects with LPR	25	11.8

Variable	Mean	SD
Age	23.92	6.620
RSI score	10.50	9.085
SFAR score	6.72	3.560
SFAR score in subjects with AR	8.08	3.290
RSI score in subjects with LPR	13.00	9.705
SFAR score in asthmatic subjects	10.50	2.841
SFAR score in non-asthmatic subjects	6.45	3.455
RSI score in asthmatic subjects	16.14	11.645
RSI score in non-asthmatic subjects	10.11	8.756

SFAR: score for allergic rhinitis; RSI: reflux symptom index; LPR: laryngopharyngeal reflux; AR: allergic rhinitis; SD: standard deviation.

**Table 2:** Comparing positive SFAR score with positive RSI score.

Characteristic	Positive LPR symptoms	Percentage (CI 95%)	Negative LPR symptoms	Percentage (CI 95%)	P value	OR
**SFAR score**						
Positive allergic rhinitis	141	66.5	200	43.4	< 0.000001	2.592 (1.846–3.639)
Negative allergic rhinitis	71	33.5	261	56.6		
**SFAR score in subjects with asthma**						
Positive allergic rhinitis	22	88.0	16	84.2	*NS*	1.375 (0.245–7.717)
Negative allergic rhinitis	3	12.0	3	15.8		
**SFAR score in subjects without asthma**						
Positive allergic rhinitis	119	63.6	184	41.6	< 0.000001	2.454 (1.724–3.492)
Negative allergic rhinitis	68	36.4	258	58.4		

CI: Confidence interval; SFAR: Score for allergic rhinitis; RSI: Reflux symptom index; OR: Odds ratio; NS: Not significant.

Chi-square was used to determine the significance of the comparisons in this table.

Comparing each SFAR item with having LPR using chi-square and odds-ratio is demonstrated in (Table 3). Having symptoms suggestive of LPR was significantly associated with AR symptoms of sneezing, runny nose, blocked nose, nasal symptoms with itchy eyes, time of occurrence, triggers, perceived allergic status, previous medical diagnosis, familial history of allergy and father history of allergy p < 0.05. When excluding subjects with asthma, LPR was still significantly associated with AR p < 0.0001 (OR, 2.454; 95% CI 1.724–3.492). However, if we only included subjects with asthma, no significant association was found p > 0.05 (Table 2). Having asthma was associated with LPR symptoms p = 0.0002 (OR 3.096; 95% CI 1.665–5.759) and AR p < 0.0001 (OR 6.772; 95% CI 2.823–16.248).

**Table 3:** Comparing each SFAR item with having positive or negative LPR according to RSI.

SFAR items	Positive RSI	Percentage	Negative RSI	Percentage	P value	OR (CI = 95%)
**Sneezing**						
Negative	91	42.9	267	57.9	0.0003	1.830 (1.317–2.543)
Positive	121	57.1	194	42.1		
**Runny nose**						
Negative	88	41.6	234	50.8	0.026	1.453 (1.046–2.018)
Positive	124	58.4	227	49.2		
**Blocked nose**						
Negative	63	29.7	240	52.1	< 0.0001	2.568 (1.816–3.632)
Positive	149	70.3	221	47.9		
**If yes for any of the previous, has this problem been accompanied with itchy eyes**						
Negative	87	41.0	278	60.3	< 0.0001	2.183 (1.567–3.040)
Positive	125	59.0	183	39.7		
**In which of the past 12 months (or in which season) did this nose problem occur**						
Unspecified	100	47.2	265	57.5	0.004^a^	1.514 (1.092–2.100)^a^
Pollen season	30	14.2	76	16.5		
Perennial	82	38.7	120	26.0		
**What trigger factors provoke or increase your nose problem?**						
None	57	26.9	189	41.0	0.002^b^	1.890 (1.324–2.697)
Animals	2	0.9	4	0.9		
Pollens, house dust (mites), dust	130	61.3	241	52.3		
All of the above	23	10.8	27	5.9		
**Do you think to be allergic?**						
Negative	81	38.2	242	52.5	< 0.001	1.787 (1.282–2.491)
Positive	131	61.8	219	47.5		
**Have you been tested for allergy (SPT, IgE)?**						
Negative	190	89.6	425	92.2	*NS*	1.367 (0.783–2.387)
Positive	22	10.4	36	7.8		
**If yes what was the result**						
Negative	9	45.0	16	47.1	*NS*	1.086 (0.358–3.293)
Positive	11	55.0	18	52.9		
**Has a doctor already diagnosed that you suffer/suffered from asthma, eczema, or allergic rhinitis?**						
Negative	96	45.3	309	67.0	< 0.0001	2.456 (1.761–3.427)
Positive	116	54.7	152	33.0		
**Is any member of your family suffering from asthma, eczema, or allergic rhinitis?**						
Negative	74	34.9	207	44.9	0.015	1.520 (1.085–2.128)
Positive	138	65.1	254	55.1		

Chi-square was used to determine the significance of the comparisons in this table.

SFAR: Score for allergic rhinitis; RSI: Reflux symptom index; OR: Odds ratio; CI: confidence interval; SPT: skin prick testing; IgE: immunoglobulin E.

^a^OR was calculated between unspecific and specific with P = 0.013.

^b^OR was calculated between none and other variables with P < 0.001.

**Figure 1:**
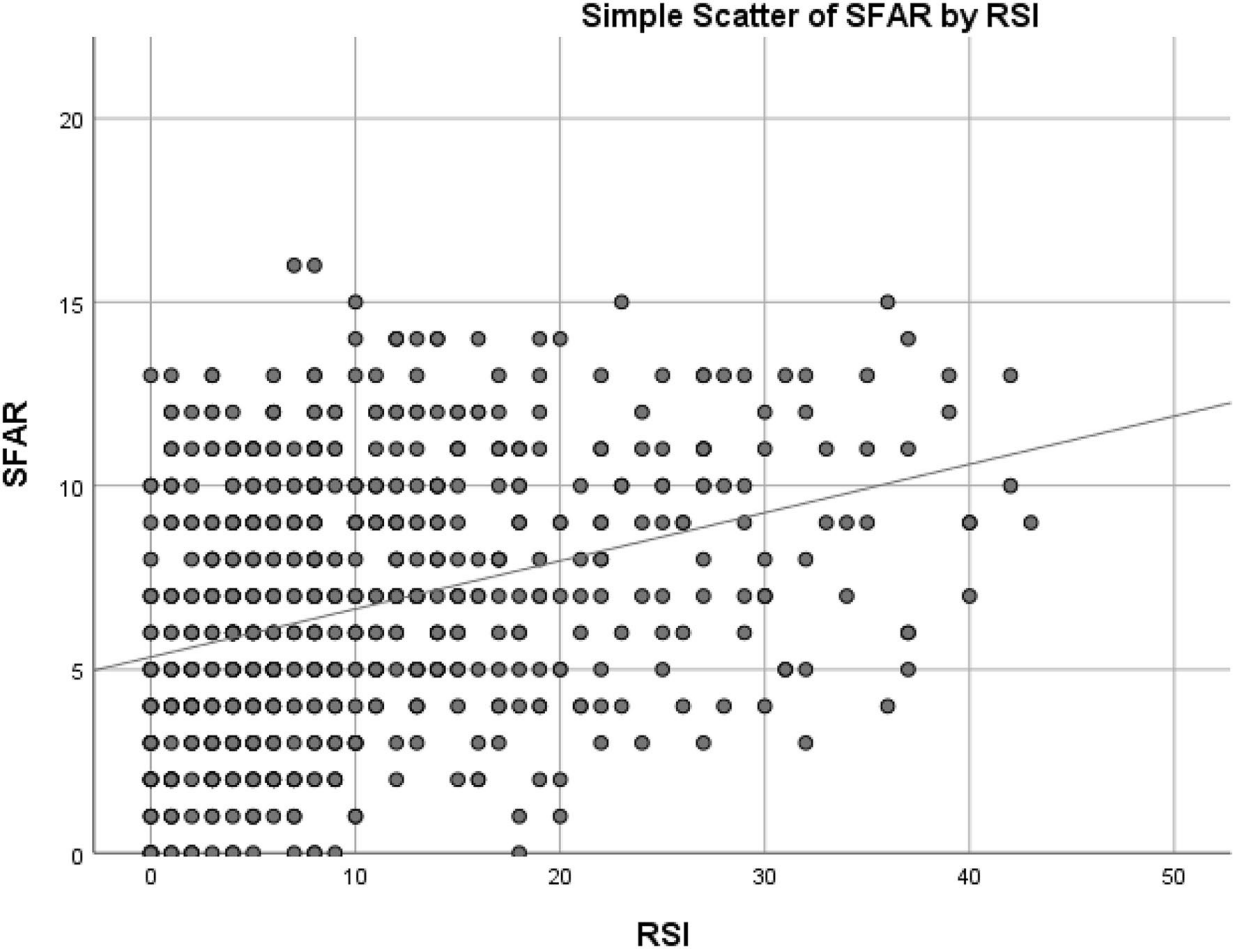
Showing the scatter of RSI and SFAR score values in all participants with r = 0.334 at p < 1 × 10^–19^.

**Figure 2:**
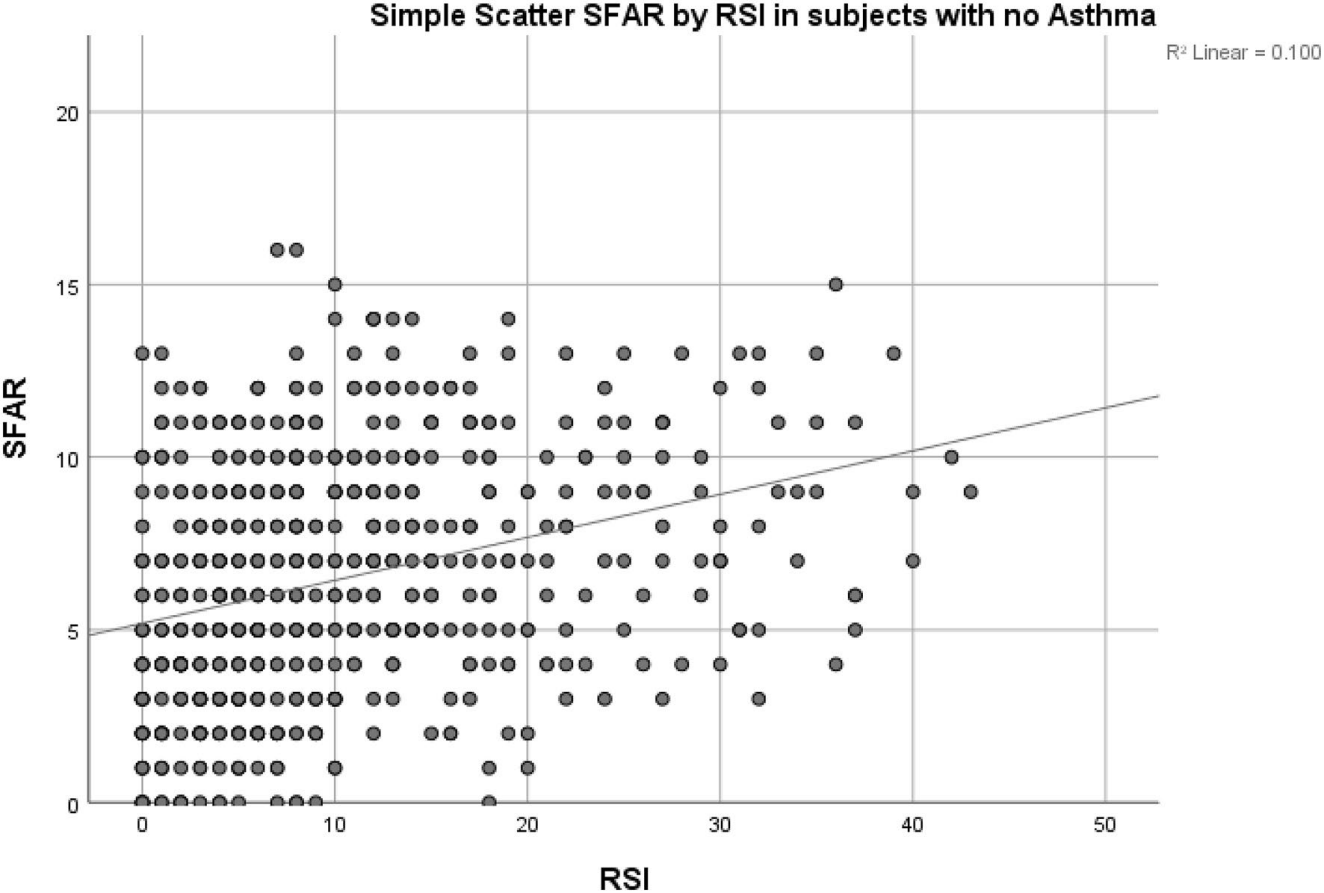
Showing the scatter of RSI and SFAR score values in subjects when excluding asthma with r = 0.316 at p < 1 × 10^–19^.

When bivariate Pearson correlation was used to compare the scores of SFAR and RSI, a significant moderate correlation was found r = 0.334 (Fig. 1) with p < 1 × 10^–19^. Another significant moderate correlation was found r = 0.316 when excluding asthma p < 1 × 10^–19^ (Fig. 2). However, in subjects who have asthma, no significant correlation was found when comparing scores p > 0.05. Comparing between each RSI item and having AR or not using independent t-test is demonstrated in Table 4. AR symptoms is associated with each LPR symptom as demonstrated in (Table 4) (p < 0.001). Having asthma was associated with higher RSI and SFAR scores (p < 0.001).

**Table 4:** Comparing each RSI item with subjects with positive and negative SFAR.

RSI items	Mean scores in subjects with positive SFAR ± SD	Mean score (CI 95%)	Mean scores in subjects with negative SFAR ± SD	Mean score (CI 95%)	P value
Sore throat	1.04 ± 1.302	0.90–1.18	0.66 ± 1.056	0.55–0.78	< 0.001
Sputum production	1.62 ± 1.544	1.47–1.79	0.98 ± 1.200	0.86–1.11	< 0.001
Excessive secretions	1.67 ± 1.586	1.49–1.84	0.84 ± 1.226	0.71–0.97	< 0.001
Dysphagia	1.10 ± 1.416	0.96–1.26	0.57 ± 1.090	0.46–0.70	< 0.001
Coughing after eating, sleeping, or lying down	1.41 ± 1.587	1.25–1.57	0.98 ± 1.409	0.84–1.14	< 0.001
Breathing difficulties	1.38 ± 1.529	1.22–1.54	0.71 ± 1.189	0.59–0.84	< 0.001
Extreme coughing episodes	1.37 ± 1.594	1.20–1.56	0.94 ± 1.464	0.79–1.11	< 0.001
A sense of foreign body in throat	1.46 ± 1.552	1.30–1.62	0.84 ± 1.248	0.70–0.98	< 0.001
Epigastric burning sense, chest pain, indigestion, and GORD	1.96 ± 1.732	1.78–2.14	1.41 ± 1.467	1.25–1.56	< 0.001
Total score	13.00 ± 9.705	11.93–14.04	7.93 ± 7.599	7.19–8.76	< 0.001

Independent t-test was used to determine the significance of the comparisons in this table.

CI: confidence interval; SFAR: score for allergic rhinitis; RSI: reflux symptom index; SD: standard deviation; GORD: gastro-oesophageal reflux disease.

RSI mean score was 10.50 ± 9.085 (CI 95%: 9.83–11.25) and SFAR mean score was 6.72 ± 3.560 (95% CI 6.45, 7.00). SFAR, and RSI scores according to one another and to asthma are demonstrated in (Table 1).

## Discussion

### AR and LPR association

RSI score in our study was significantly correlated with SFAR score. This was also evident when comparing the results of these two scales as having LPR symptoms was associated with AR symptoms. This was also found when comparing each symptom of LPR with having AR and vice versa. Having either LPR or AR was associated with a 2.6-fold increase in the in risk of having the other. The strength of the correlation between RSI and SFAR scores was moderate in strength (r = 0.334) and demonstrated in (Fig. 1).

This association between LPR and AR in our study is similar to other studies^22,23^. AR and allergic laryngitis (AL) can have similar manifestations^32^. Epigastric burning sensation, chest pain, and indigestion were the most common symptoms of LPR in Syria while having a sore throat was the least common^4^. AR symptoms can include sneezing, nasal itching, rhinorrhoea, and congestion^19,20^. As AR/AL and LPR can have overlapping symptoms, distinguishing them was proven difficult in one small study^19^. However, AR and LPR symptoms can overlap and the most common symptoms would be repeated throat cleaning, and a globus sensation^22^.

The frequent swallowing with AR is due to the itching sensation and post-nasal drip. This frequent swallowing would also increase due to the reflux. Furthermore, nasal mucosa will be affected by AR which leads to LPR as AR causes the mucosa to be congested, oedematous, and increases mucous secretions^23^. This common effect on the mucosa could explain the association between LPR and AR.

This association could also be explained similarly to the explanation of GORD and AR as they are among the main causes of chronic cough; moreover, increased reoccurrence of the cough with reflux symptom has been reported in patients with GORD and in those without symptoms of GORD who have rhinitis, indicating that other factors contribute to the development of chronic cough. In addition, the coexistence of GORD and chronic rhinosinusitis (CRS) were reported by multiple studies^21–23,33–35^. One theory explaining this phenomena was that *Helicobacter pylori*, which is usually found in the gastric mucosa and promotes heartburning, could exist in the sinonasal cavity^36^. Another theory associated GORD with bronchial spasm^34,37^. However, one study suggested that GORD would only worsen nasal symptom scores but did not cause chronic rhinosinusitis^34^.

However, although study data showed that placebo can be as effective as PPI therapy, empirical treatment with PPI is still recommended^38^. Nevertheless, in another study, an association between pH Ryan score and total SFAR score was found, which could be related to LPR^22^.

LPR diagnosis can be much more complicated with many methods from interviews to challenging treatment methods as LPR has many vague and unspecific symptoms such as throat clearing, globus pharyngeus, and hoarseness^25,39,40^. Many of the diagnostic methods cannot be used in Syria due to the limited resources as over 80% of the population is under poverty line and most research does not have a proper funding^41,42^. Non-instrumental methods can also be used such as RSI and the reflux finding score (RFS)^24,25^, but they cannot be used as the gold standard to diagnose LPR as they are based on symptoms.

The diagnosis of AR is based on a detailed clinical history and skin prick test. Serum specific IgE to the whole allergen extracts or components could be used as second- and third-line tests, respectively. The association between the clinical evaluation and the results of the mentioned tests is crucial for a correct diagnosis^43^. This can justify using questionnaire-based tools as their accuracy is acceptable and much cheaper than diagnostic tests, especially in developing countries.

### Asthma

We found that having AR and LPR symptoms were significantly associated with having asthma. The association between AR and LPR persisted when including subjects with no asthma. However, there was no association between AR and LPR when only including subjects with asthma, but our study only had 44 subjects with asthma which might not have detected the association. Our study found that having either asthma or AR was associated with a 6.8-fold increase in the incidence of the other. We also found that having either asthma or LPR was associated with a 3.1-fold increase in the incidence of the other. A study in Syria found that asthma, allergies, and respiratory conditions were associated with having LPR symptoms^13^.

As the larynx exists in a critical location that connects upper and lower airways which have a similar microscopic structure, it is suggested that having a disease in one portion of this system would affect the entire respiratory system^44^. One study found that about 25% of patients who had AR also had asthma. Having asthma was also associated with having a much higher incidence of AR^45^. Several previous studies found that having AR or asthma was associated with a threefold increase of having the other and that AR diagnosis was mostly made before asthma presentation^46,47^.

Socioeconomic status was not studied as it is hard to accurately determine the financial situations in Syria due to rapid changing, different living expenses, and asking directly for month salary being inappropriate^41,48^.

### Limitation

We do acknowledge that our study has many limitations that need to be addressed. The small sample size, for asthma in particular, could be limiting. No clinical diagnosis was made, and only self-reported methods were used which may overestimate the symptoms and render the answers to be subjective. Subjects who do not have online access could not participate. We could not target the population at risk. This study included more females than males and included relatively young participants which may affect the generalizability of the results. The cross-sectional method is also limiting as causality cannot be determined.

The common symptoms of the aerodigestive tract can be misleading and misdiagnosed as either AR or LPR. Moreover, smoking, asthma, mental distress, and allergies may cause the same symptoms by various ways. In Syria, LPR had a prevalence of 31.9% and AR had a prevalence of 47.9%. They were both associated with asthma, allergies, distress from war, and smoking^4,13^. Moreover, 61.2% of Syrians had moderate to severe mental distress, and 60.8% had symptoms of post-traumatic distress disorder (PTSD)^41^. School students were also affected as 53% had PTSD symptoms and 62.2% had problematic anger^48^. All the previous factors can contribute to the high prevalence of symptoms of LPR and AR. We need more studies to accurately determine these associations as LPR and AR have many common factors that can be confounding when detecting their association. Finally, there is a lack of studies about many medical conditions and risk factors in Syria. Furthermore, war and the unique environment of Syria impose different risk factors to many medical conditions which can also be the case for LPR and AR^42,49^.

*In conclusion*, many studies had contradicting data about LPR and AR as their definition and methods of diagnosis may differ and overlap. LPR, AR, and asthma are significantly associated with one another which may be attributed to common symptoms and aetiologies. Our study found that having symptoms suggestive of LPR was associated with having AR (OR = 2.6), and a significant positive correlation was found when comparing their scores (r = 0.334). Having asthma was associated with LPR symptoms (OR = 3.1) and AR (OR = 6.8). We need more detailed methods for diagnosis of AR and LPR which both have high prevalence, and better management of these conditions may improve the quality of life for a very large population for years.

## References

[1] Koufman JA (2016). Laryngopharyngeal reflux: Position statement of the committee on speech, voice, and swallowing disorders of the American Academy of Otolaryngology-Head and Neck Surgery. Otolaryngol. Head Neck Surg..

[2] Koufman JA, Amin MR, Panetti M (2016). Prevalence of reflux in 113 consecutive patients with laryngeal and voice disorders. Otolaryngol. Head Neck Surg..

[3] Havemann BD, Henderson CA, El-Serag HB (2007). The association between gastro-oesophageal reflux disease and asthma: A systematic review. Gut.

[4] Kakaje A (2020). Laryngopharyngeal reflux in war-torn Syria and its association with smoking and other risks: An online cross-sectional population study. BMJ Open.

[5] Castell DO, Schnatz PF (1995). Gastroesophageal reflux disease and asthma. Chest.

[6] Field SK (2002). Asthma and gastroesophageal reflux. Chest.

[7] Choy D, Leung R (1997). Gastro-oesophageal reflux disease and asthma. Respirology.

[8] Zerbib F (2002). Effects of bronchial obstruction on lower esophageal sphincter motility and gastroesophageal reflux in patients with asthma. Am. J. Respir. Crit. Care Med..

[9] Ates F, Vaezi MF (2014). Insight into the relationship between gastroesophageal reflux disease and asthma. Gastroenterol. Hepatol..

[10] Dykewicz MS, Fineman S (1998). Executive summary of joint task force practice parameters on diagnosis and management of rhinitis. Ann. Allergy Asthma Immunol..

[11] Bernstein JA (2010). Allergic and mixed rhinitis: Epidemiology and natural history. Allergy Asthma Proc..

[12] Abdulrahman H (2012). Nasal allergies in the middle eastern population: Results from the “Allergies in Middle East Survey”. Am. J. Rhinol. Allergy.

[13] Kakaje A (2020). Allergic rhinitis and its epidemiological distribution in Syria: A high prevalence and additional risks in war time. Biomed. Res. Int..

[14] Nathan RA (2007). The burden of allergic rhinitis. Allergy Asthma Proc..

[15] Woolcock AJ (2001). The burden of asthma in Australia. Med. J. Aust..

[16] Beasley R, The International Study of Asthma and Allergies in Childhood (ISAAC) Steering Committee (1998). Worldwide variation in prevalence of symptoms of asthma, allergic rhinoconjunctivitis, and atopic eczema: ISAAC. Lancet.

[17] Trikojat K (2017). Memory and multitasking performance during acute allergic inflammation in seasonal allergic rhinitis. Clin. Exp. Allergy.

[18] Khan DA (2014). Allergic rhinitis and asthma: Epidemiology and common pathophysiology. Allergy Asthma Proc..

[19] Randhawa P, Mansuri S, Rubin J (2009). Is dysphonia due to allergic laryngitis being misdiagnosed as laryngopharyngeal reflux?. Logopedics Phoniatrics Vocol..

[20] Turley R (2011). Role of rhinitis in laryngitis: Another dimension of the unified airway. Ann. Otol. Rhinol. Laryngol..

[21] Katle E-J, Hatlebakk JG, Steinsvåg S (2013). Gastroesophageal reflux and rhinosinusitis. Curr. Allergy Asthma Rep..

[22] Alharethy S (2018). Correlation between allergic rhinitis and laryngopharyngeal reflux. Biomed. Res. Int..

[23] Kung Y-M (2019). Allergic rhinitis is a risk factor of gastro-esophageal reflux disease regardless of the presence of asthma. Sci. Rep..

[24] Belafsky PC, Postma GN, Koufman JA (2001). The validity and reliability of the reflux finding score (RFS). Laryngoscope.

[25] Belafsky PC, Postma GN, Koufman JA (2002). Validity and reliability of the reflux symptom index (RSI). J. Voice.

[26] Wise SK, Wise JC, DelGaudio JM (2016). Gastroesophageal reflux and laryngopharyngeal reflux in patients with sleep-disordered breathing. Otolaryngol. Head Neck Surg..

[27] Kelchner LN (2007). Reliability of speech-language pathologist and otolaryngologist ratings of laryngeal signs of reflux in an asymptomatic population using the reflux finding score. J. Voice.

[28] Farahat M, Malki KH, Mesallam TA (2012). Development of the Arabic version of reflux symptom index. J. Voice.

[29] Annesi-Maesano I (2002). The score for allergic rhinitis (SFAR): A simple and valid assessment method in population studies. Allergy.

[30] Alharethy S (2017). Validation of the Arabic version of the score for allergic rhinitis tool. Ann. Saudi Med..

[31] Kakaje, A., Alhalabi, M. M., Alyousbashi, A., Ghareeb, A. Allergic rhinitis, asthma and gastro-esophageal reflux disease: a cross-sectional study on their reciprocal relations. Preprint at 10.21203/rs.3.rs-29393/v1 (2020). 10.1038/s41598-020-80793-1 PMC7858587 33536455

[32] Krouse JH, Altman KW (2010). Rhinogenic laryngitis, cough, and the unified airway. Otolaryngol. Clin. N. Am..

[33] García-Compeán D (2000). Prevalence of gastroesophageal reflux disease in patients with extraesophageal symptoms referred from otolaryngology, allergy, and cardiology practices: A prospective study. Dig. Dis..

[34] Hanna BC, Wormald PJ (2012). Gastroesophageal reflux and chronic rhinosinusitis. Curr. Opin. Otolaryngol. Head Neck Surg..

[35] Loehrl TA, Smith TL (2004). Chronic sinusitis and gastroesophageal reflux: Are they related?. Curr. Opin. Otolaryngol. Head Neck Surg..

[36] Zdek A (2003). A possible role of helicobacter pylori in chronic rhinosinusitis: A preliminary report. Laryngoscope.

[37] Wong IWY (2010). Gastroesophageal reflux disease and chronic sinusitis: In search of an esophageal–nasal reflex. Am. J. Rhinol. Allergy..

[38] Bytzer P (2008). Management of laryngopharyngeal reflux with proton pump inhibitors. Ther. Clin. Risk Manag..

[39] Pribuisiene R, Uloza V, Jonaitis L (2002). Typical and atypical symptoms of laryngopharyngeal reflux disease. Medicina.

[40] Oelschlager BK (2005). Typical GERD symptoms and esophageal ph monitoring are not enough to diagnose pharyngeal reflux. J. Surg. Res..

[41] Kakaje, A. *et al.**Mental disorder and PTSD in Syria during wartime: a nationwide crisis* (2020). 10.1186/s12888-020-03002-3 PMC7778805 33388026

[42] Kakaje A (2020). Rates and trends of childhood acute lymphoblastic leukaemia: An epidemiology study. Sci. Rep..

[43] Ansotegui IJ (2020). IgE allergy diagnostics and other relevant tests in allergy, a World Allergy Organization position paper. World Allergy Organ. J..

[44] Krouse JH (2008). The unified airway—Conceptual framework. Otolaryngol. Clin. N. Am..

[45] Compalati E (2014). The link between allergic rhinitis and asthma: The united airways disease. Expert Rev. Clin. Immunol..

[46] Huovinen E (1999). Incidence and prevalence of asthma among adult Finnish men and women of the Finnish twin cohort from 1975 to 1990, and their relation to hay fever and chronic bronchitis. Chest.

[47] Settipane RJ, Hagy GW, Settipane GA (1994). Long-term risk factors for developing asthma and allergic rhinitis: A 23-year follow-up study of college students. Allergy Asthma Proc..

[48] Kakaje A (2020). Post-traumatic stress disorder (PTSD), anger and mental health of school students in Syria after nine years of conflict: A large-scale school-based study. Pyschol. Med..

[49] Al Habbal, A. *et al.* Risk factors associated with epilepsy in children and adolescents: A case-control study from Syria. *Epilepsy Behav.* (2020). 10.1016/j.yebeh.2020.107596 33246894

